# Transgender Transitioning and Change of Self-Reported Sexual Orientation

**DOI:** 10.1371/journal.pone.0110016

**Published:** 2014-10-09

**Authors:** Matthias K. Auer, Johannes Fuss, Nina Höhne, Günter K. Stalla, Caroline Sievers

**Affiliations:** 1 Department of Internal Medicine, Endocrinology and Clinical Chemistry, Max Planck Institute of Psychiatry, Munich, Germany; 2 Institute for Sex Research and Forensic Psychiatry, Center for Psychosocial Medicine, University Medical Center Hamburg-Eppendorf, Hamburg, Germany; 3 Department Molecular Psychology, Max Planck Institute of Psychiatry, Munich, Germany; Claremont Colleges, United States of America

## Abstract

**Objective:**

Sexual orientation is usually considered to be determined in early life and stable in the course of adulthood. In contrast, some transgender individuals report a change in sexual orientation. A common reason for this phenomenon is not known.

**Methods:**

We included 115 transsexual persons (70 male-to-female “MtF” and 45 female-to-male “FtM”) patients from our endocrine outpatient clinic, who completed a questionnaire, retrospectively evaluating the history of their gender transition phase. The questionnaire focused on sexual orientation and recalled time points of changes in sexual orientation in the context of transition. Participants were further asked to provide a personal concept for a potential change in sexual orientation.

**Results:**

In total, 32.9% (n =  23) MtF reported a change in sexual orientation in contrast to 22.2% (n =  10) FtM transsexual persons (p =  0.132). Out of these patients, 39.1% (MtF) and 60% (FtM) reported a change in sexual orientation before having undergone any sex reassignment surgery. FtM that had initially been sexually oriented towards males ( = androphilic), were significantly more likely to report on a change in sexual orientation than gynephilic, analloerotic or bisexual FtM (p  =  0.012). Similarly, gynephilic MtF reported a change in sexual orientation more frequently than androphilic, analloerotic or bisexual MtF transsexual persons (p  =  0.05).

**Conclusion:**

In line with earlier reports, we reveal that a change in self-reported sexual orientation is frequent and does not solely occur in the context of particular transition events. Transsexual persons that are attracted by individuals of the opposite biological sex are more likely to change sexual orientation. Qualitative reports suggest that the individual's biography, autogynephilic and autoandrophilic sexual arousal, confusion before and after transitioning, social and self-acceptance, as well as concept of sexual orientation itself may explain this phenomenon.

## Introduction

Western sexual norms that were established during the modern era based identity upon gender-sex identity and sexual desires e.g. homosexual, bisexual or heterosexual identity [1], [2]. Sexual identity was seen as something ingrained in the psychological and biological makeup of an individual [3]. However, among other societies notions of identity based on sexual behavior were absent or emerged only in the course of colonialism [3]. Sexual identity and the concept of *the heterosexual* and *the homosexual* are thus categories that follow Western notions of an identity based on sexual desire or acts.

Sexual orientation is highly influenced by the concept of a sexual identity and refers to “*an enduring pattern of emotional, romantic, and/or sexual attractions to men, women, or both sexes*” [4]. Sexual orientation is determined in early life and usually unchangeable thereafter [5], [6] although some lesbian women and gay men come out later in life. Categories of sexual orientation and changes in sexual orientation can only be categorized because of the notion of a stable sexual identity that is ingrained in the individual.

However, it has been reported that transgender individuals report a change in their sexual orientation over time [7]–[9]. Here we aim to categorize sexual orientation in a transsexual population and study changes in self-reported sexual orientation. Participants are categorized into four groups as initially suggested by Magnus Hirschfeld [10], based on their erotic interest: gynephilic  =  attracted to women, androphilic  =  attracted to men, bisexual  =  attracted to both men and women, and analloerotic  =  not attracted to other people. Reported changes in sexual attraction or self-defined sexual orientation were linked to the gender transition process before [11]. Meier and colleagues demonstrated that 40 % of female-to-male (FtM) transsexual persons report changes in sexual orientation in the course of their lives and this may partially be attributed to the initiation of testosterone therapy [12]. In male-to-female (MtF) transsexual persons a significant change in sexual orientation has also been reported especially after the transition process [11]. Whether changes in sexual orientation were attributable to biological effects of hormone therapy or to psychological factors and how to interpret these self-reported changes is still under debate.

In summary, it is unclear if sexual orientation of transsexual persons (i) changes in the course of the transition period e.g. after introduction of hormone therapy or (ii) is indeed unchangeable and only refers to a psychological interpretation that is related to transition in self-reported sexual orientation.

We therefore aimed to systematically evaluate changes in sexual orientation in a transsexual population in regard to the chronology of important life events of the transition such as start of so-called ”real-life experience”, initiation of hormonal therapy or performed sex reassignment surgery by combining quantitative and qualitative data. We studied how frequently changes in sexual orientation are reported and which of the aforementioned variables may possibly correlate with changes in sexual orientation in transsexual persons. By this approach we were ultimately aiming to identify the influence of important life events on self-reported sexual orientation.

## Methods

### Cohort

We included 115 (70 MtF and 45 FtM) transsexual, caucasian patients into this study who visited the endocrine outpatient clinic of the Max Planck Institute of Psychiatry, Munich, between May 2011 and February 2012, either for initial endocrinological evaluation or follow-up examination. After giving written informed consent, they were asked to complete a questionnaire, retrospectively evaluating the history of their gender transition phase. Assessment procedures were monitored by a quality circle in Munich, where experts regularly meet to discuss questions related to transsexualism. Inclusion criteria were age over 18 years, diagnosis of gender identity disorder (GID) according to DSM-IV criteria [13] assessed by a mental health professional and absence of any disorder of sexual development (DSD). The study was approved by the local Ethical committee of the Ludwig Maximilians University in Munich.

### Questionnaire on transition phase, diagnostic and therapeutic process and sexual orientation

Data in this study were assessed by means of a self-report questionnaire. Questions included recalled age of first signs of cross-sex gender feelings (“when was the first time that you became aware about your cross-sex gender identification”), further referred to as age of onset.

Further variables were: time point of revealing their gender identity for the first time to a family member or friend, initiation of cross sex hormone treatment (CSHT), date of first sex reassignment surgery, date of starting “real-life experience”, date of first contact with a mental health practitioner as part of the transition process, respectively time point of official clinical GID diagnosis. Exact dates of first psychological counseling, start of “real-life experience”, cross-hormone-treatment and date and kind of sex reassignment surgery were also verified by the patient's files.

The section on sexual orientation included questions on current sexual orientation (“What is your current sexual preference?”), occurrence (“Have you ever experienced a change in your sexual orientation?”), recalled time-point (“Could you please guess when approximately such a change evolved?”) and kind of change in sexual attraction where participants had to choose between: attraction to females (gynephilic), attraction to males (androphilic), attraction to both (bisexual) or attraction to neither of those (further referred to as analloerotic) according to the classification of sexual orientation by the DSM-IV-TR specifiers for GID diagnosis [14]. They further had the opportunity to specify their sexual orientation.

In case of reported change in sexual orientation, participants were further asked to define the kind of change (e.g. from males towards females etc.) and if there had been multiple shifts in this regard.

Furthermore, they were asked to describe the approximate time point of a potential change in sexual orientation allowing to arrange changes of sexual orientation chronologically in regard to particular transition time events, namely date of first counseling with a mental health practitioner as part of the transition process, initiation of cross sex hormone treatment, first sex reassignment surgery, self-reported time point of officially living in the new gender role.

In the qualitative part of this study, participants were asked to comment and provide a personal explanation as a free text on this change.

### Statistical analyses

All statistical analyses were performed using SPSS v.18.0 (IBM Corp., Armonk, NY, USA). A two-tailed p-value of 0.05 and lower was considered statistically significant with a 95% confidence interval (CI). The Fisher's exact test was used to compare dichotomous variables. Independent Student's t-test was used to compare means of continuous variables. One-way analysis of variance (ANOVA) was used for comparison of more than two groups such as sexual orientation (androphilic, gynephilic, bisexual, analloerotic) in variables representing the history of transition (age of self-aware, age of psychological counseling, age of “real-life experience”, age of hormone-therapy, age of sex reassignment surgery, age when sexual orientation changed). For post-hoc comparisons Fisher's LSD (Least Significant Difference) was applied.

## Results

### Initial self-reported sexual orientation

Basic characteristics of our study sample can be found in Table 1. In MtF, 25.7 % of participants indicated that they initially had been sexually attracted to males ( =  androphilic) and 51.4 % to females ( =  gynephilic). Bisexuality was reported by 10 % and 12.9 % declared themselves as having been attracted to neither sex ( =  analloerotic). Most FtM transsexual persons had been gynephilic (73.4 %) while six individuals (13.3 %) indicated having been sexual orientated towards males ( = androphilic). Bisexuality was reported by 8.8 % and one participant (2.2 %) had described himself as analloerotic. No subject reported on sexual orientation towards other transsexual persons.

**Table 1:** Basic characteristics at the time of evaluation.

	MtF		FtM			p-value
	N	%	N		%	
**variable (mean ± SD (range))**						
**sex**	70	60.9	45		30.1	
**age**	46.7 ± 13.4 (18–80)		37 ± 9.8 (19–60)			<0.001†
**cross-hormone treatment**						
yes	63	90	42		93.3	n.s.§
no	7	10	3		6.7	
Testosterone (intramuscular)			32		78.0	
Testosterone (transdermal)			9		22.0	
Estradiol(oral)	9	14.5				
Estradiol(transdermal)	30	48.4				
Estradiol + Antiandrogen (transdermal)	14	22.6				
Estradiol + Progesterone	9	14.5				
sex reassignement surgery*						
any SRS	40	57.1	27		60.0	n.s.§
any genital surgery	39	55.7	26		57.8	
*Only hysterectomy/ovariectomy/mastectomy*			16		33.6	
*+ phalloplasty*			10		22.2	
*mastectomy only*			1		2.2	
*breast augmentation only*	1	1.4				
No SRS	30	42.9	18		40.0	
**age of onset**	10.8 ± 8.2 (3–49)		8.2 ± 6.5 (2–35)			n.s.†
before or at the age of 12	43	68.3	32		82.1	n.s.§
after the age of 12	20	31.7	7		17.9	
	N					
	Age (in years)					
first psychological counseling	38.1 ± 13.3 (11–70)		29.2 ± 8.5 (17–48)			<0.001†
start of „real-life experience“	38.1 ± 10.9 (18–70)		29.2 ± 8 (18–47)			<0.001†
cross-sex hormone treatment	38.6 ± 14 (16–69)		31.1 ± 8.5 (17–50)			0.003†
sex reassignment surgery	42.4 ± 12.5 (18–70)		32.8 ± 9 (20–56)			0.001†

FtM: hysterectomy, ovariectomy, mastectomy, phalloplasty; MtF: bilateral orchidectomy, penectomy, breast augmentation if necessary, vaginoplasty, vulvoplasty, SD  =  standard deviation, n.s.  =  not significant on a significance level of p≤.05.

† student's t-test.

§ Fisher's exact test.

### Initial self-reported sexual orientation and history of transition

In MtF, androphilic participants were also younger when they started their „real-life experience “in the new gender role (p<0.01), started cross-sex hormone treatment (p<0.01) and underwent first sex reassignment surgery (p<0.01). Bisexual MtF reported on a significantly older age of onset, younger age when starting their “real-life experience” and start of cross-sex hormone treatment than gynephilic MtF (all p<0.05) (Table 2).

**Table 2:** Initial sexual orientation and history of transition in MtF.

	MtF			
	androphilic	gynephilic	bisexual	analloerotic
	N = 18 (25.7%)	N = 36 (51.4%)	N = 7 (10%)	N = 9 (12.9%)
age	41.6(16.4)^a^	51(9.6)^a,b^	36(10.8)^b^	47.9(15.9)
**Mean age of**				
onset	7.2(3.9)	11.6(9.9)	11.7(7.3)	14(5.8)
first psychological counseling	32(13.8)^c,i^	42.6(11.5)^c^	31.3(8.7)^i^	39(17)
start of „real-life experience“	36.4(10.8)^d^	45.2(9.6)^d,e^	33(7.7)^e^	40.7(12.1)
CSHT	31.1(13.8)^f^	42.8(11.8)^f,g^	31.9(9.5)^g^	41.1(17)
SRS	35.3(14.1)^h^	47.5(10.3)^h^	36.2(9.3)	34(12.7)
Change in SO (N = 23)	32.4(18)	40.8(7.6)	-	28(19.8)

a,b,c,f,h p <0.01.

d,e,g,i p <0.05.

(ANOVA).

CSHT: Cross-sex hormone treatment.

SRS: Sex reassignment surgery.

SO: Sexual orientation.

a-h mean significant differences between those two groups, e.g. a means a significant difference (p <0.01) between androphilic and gynephilic MtF in terms of age.

In FtM, significant differences between androphilic and gynephilic FtM were seen in the variables age of onset, first psychological counseling and start of cross-sex hormone treatment (all p<0.05). There were no further significant differences regarding other sexual orientations and any variable of transition history (Table 3).

**Table 3:** Initial sexual orientation and history of transition in FtM.

	FtM			
	androphilic	gynephilic	bisexual	analloerotic
	N = 6	N = 33	N = 4	N = 2
	(13.3%)	(73.4%)	(8.8%)	(4.4%)
age	44.3(9.2)	35.9(9.3)	39.3(12.9)	28.5(10.6)
**Mean age of**				
onset	4 (4.5)^a^	7.7 (4.9)^a^	19.3 (14.3)	2
first psychological counseling	36.5 (8.7)^b^	28.1 (8.2)^b^	30.3(8.2)	24 (4.2)
start of „real-life experience“	32.8(8.1)	28(7.8)	30.7(10.7)	22.5(6.4)
CSHT	37.3(9.3)^c^	29.5(8.2)^c^	34.7(6.1)	27
SRS	38(12.3)	31.3(8.5)	39.5(2,1)	27
Change in SO (N = 10)	30.8(7.4)	33.7(14.8)	–	–

a,b,c p <0.05.

CSHT: Cross-sex hormone treatment.

SRS: Sex reassignment surgery.

SO: Sexual orientation.

a-c mean significant differences between those two groups, e.g. “a” means a significant difference (p <0.05) between androphilic and gynephilic FtM in terms of age of onset.

### Change in self-reported sexual orientation

About one third of MtF (32.9 %, N  =  23) reported a change in sexual orientation during their life, in contrast to 22.2 % (N  =  10) in the FtM group (n.s.). Nine MtF (39.1 % of all that reported a changed sexual orientation (N = 23)) and six FtM (60 % of all that changed sexual orientation (N = 10)) reported a change in sexual orientation without or before any surgical treatment. Out of these, four MtF (17.4 % of all that reported a changed sexual orientation) and 2 FtM (20 % of all that reported a changed sexual orientation) reported a change in sexual orientation, without or before having received cross-sex hormone treatment. Six MtF (26.1 % of all that reported a changed sexual orientation) and two FtM (20 % of all that reported a changed SO) reported a change in sexual orientation without/before psychological counseling. MtF and FtM did not differ significantly in the reported age of change in sexual orientation.

### Initial sexual orientation and change in self-reported sexual orientation

Change of self-reported sexual orientation in gynephilic MtF appeared in 15 participants (44.1 %), six reported a change to androphilia (16.6 %), eight to bisexuality (22.2 %) and one to analloerotica (2.7 %). In androphilic MtF, 5 participants (27.7 %) reported a change in sexual orientation to bisexuality (5.6 %) and gynephilia (11.1 %), two patients (11.1 %) did not report on direction of change (Table 4).

**Table 4:** Kind of change in sexual orientation in MtF.

	Change to					
**Initial orientation (N = 70)**	**androphilic**	**gynephilic**	**bisexual**	**analloerotic**	**unknown**	**all**
	N (%)	N (%)	N (%)	N (%)	N (%)	
**androphilic** (N = 18)	0 (0)	2 (11.1)	1 (5.6)	0 (0)	2 (11.1)	5 (27.7)
**gynephilic** (N = 36) ^a^	6 (16.6)	0 (0)	8 (22.2)	1 (2.7)	0 (0)	15 (41.7)
**bisexua**l (N = 7)	0 (0)	0 (0)	0 (0)	0 (0)	0 (0)	0 (0)
**analloerotic** (N = 9)	1 (11.1)	0 (0)	1 (11.1)	0 (0)	1 (11.1)	3 (33.3)
						23 (32.9)

“a” means a significant difference between gynephilic and nongynephilic MtF (p  =  0.05).

Six gynephilic FtM reported a change in sexual orientation (18.2 %) to androphilia (9.1 %) and bisexuality (9.1 %). In androphilic FtM transsexual persons, 4 reported a change in sexual orientation (66.7 %) to gynephilia. Changes in other directions were not reported (Table 5).

**Table 5:** Kind of change in sexual orientation in FtM.

	Change to					
**Initial orientation (N = 45)**	**androphilic**	**gynephilic**	**bisexual**	**analloerotic**	**unknown**	**all**
	N (%)	N (%)	N (%)	N (%)	N (%)	N (%)
**androphilic** (N = 6) a ^,^ b	0 (0)	4 (66.7)	0 (0)	0 (0)	0 (0)	4 (66.7)
**gynephilic** (N = 33) a	3 (9.1)	0 (0)	3 (9.1)	0 (0)	0 (0)	6 (18.2)
**bisexua**l (N = 4)	0 (0)	0 (0)	0 (0)	0 (0)	0 (0)	0 (0)
**analloerotic** (N = 2)	0 (0)	0 (0)	0 (0)	0 (0)	0 (0)	0 (0)
						10 (22.2)

a p  =  0.012 (X^2^-Test).

b p  =  0.001 (X^2^-Test).

“a” means a significant difference between androphilic and gynephilic FtM. “b” describes a significant difference between androphilic and nonandrophilic FtM.

Initial self-reported sexual orientation in MtF was not significantly associated with change in sexual orientation (p  =  0.05). In FtM, however, initially androphilic subjects were significantly more likely to report a change in sexual orientation than non-androphilic (gynephilic, bisexual and analloerotic, p  =  0.012) or gynephilic FtM (p  =  0.001).

### Change in self-reported sexual orientation and history of transition

Neither in MtF nor in FtM we were able to identify any variable of transition which was significantly associated with the occurrence of a reported change in sexual orientation (Table 6, Table 7).

**Table 6:** Change in Sexual orientation and basic characteristics in MtF.

	MtF						
	Change in SO			No Change in SO			
	N	%		N	%		
	23	32.9		47	67.1		
**variable**		**Mean**	**SD ±**		**Mean**	**SD±**	**p-value**
age		44.8	10.4		47.2	14.9	n.s.†
age of psychological counseling		35.2	9.0		39.4	14.8	n.s.†
age at start of „real-life experience“		38.2	8.2		41.9	12.6	n.s.†
age hormone-therapy		36.0	8.2		39.9	15.7	n.s.†
age of SRS		42.8	7.6		41.8	15.0	n.s.†
interval hormones and surgery		5.3	8.2		2.8	3.1	n.s.†
age of onset							
early onset	17			32			n.s.§
late onset	4			12			
cross-sex hormone treatment							
yes	21			40			n.s.§
no	2			5			n.s.§
SRS							
yes	16			22			n.s.§
Vagino/vulvoplasty	16			21			
breast augmentation	0			1			
no	7			23			

† student's t-test.

§ Fisher's exact test.

SRS (Sex reassignment surgery).

n.s. (not significant).

**Table 7:** Change in Sexual orientation and basic characteristics in FtM.

	FtM						
		change in SO		no change in SO			
	N	%		N	%		
	10	22.2		35	77.7		
**variable**		**Mean**	**SD ±**		**Mean**	**SD ±**	**p-value**
age		41.2	12.6		35.5	8.8	n.s.†
age of psychological counseling		32.2	10.1		28.4	7.9	n.s.†
age at start of „real-life experience“		31.1	8.1		27.3	8	n.s.†
age hormone-therapy		32.6	10.6		30.6	7.8	n.s.†
age of SRS		37.2	12.7		31.5	7.5	n.s.†
intervall hormones and sugery		2.3	2.0		1.2	0.9	n.s.†
age of onset							
early onset	8			29			n.s.§
late onset	2			6			
cross-sex hormone treatment							n.s.§
yes	10			32			
no	0			3			
SRS							
Yes total	10			35			n.s.§
Genital surgery							
hysterectomy/ovariectomy/mastectomy	6			11			
+phalloplasty	1			9			
mastectomy only	0			1			
no	3			15			

† student's t-test.

§ Fisher's exact *test*.

SRS: Sex reassignment surgery.

n.s.: not significant.

To increase homogeneity of our study population for chronological analysis, we only investigated those, who had already undergone the complete physical transition process, including cross-sex hormone treatment as well as any genital sex reassignment surgery (37 MtF and 27 FtM). In this group, sixteen MtF (43.2 %) and six FtM (22.2 %) reported changes in their sexual orientation (p  =  0.068). Only one MtF (6.7 %) and one FtM (14.3 %) reported changes in sexual orientation prior to initiation of cross-sex hormone treatment.

There was no significant difference in the direction of change in self-reported sexual orientation (e.g. from androphilic to bisexual) either by including all participants or only those having undergone cross-sex hormone treatment or sex reassignment surgery or any significant association of transition events in this subgroup (data not shown).

### Qualitative analysis

Answers given in the free text field of our questionnaire concerning change in self-reported sexual orientation and individual interpretation of this event were analyzed by two independent researchers (MKA and JF). Answers were grouped thematically. It became evident that personal explanation for a change in sexual orientation varied and ranged from explanations such as “hormone treatment increased libido” in FtM, to the statement of one MtF participants who reported that sex reassignment surgery resulted in complete loss of libido and she was therefore asexual following the procedure. Exemplary statements for each group were chosen to be reported in the discussion section and in the supplementary results (Text S1).

## Discussion

We investigated changes in self-reported sexual orientation and the relation to important life events in a large cohort of transsexual persons in Germany by means of qualitative and quantitative data. We could show that self-reported changes in sexual orientation are frequent in transsexual persons especially in originally gynephilic MtF as well as androphilic FtM. It was hypothesized before that change of sexual orientation might be influenced by hormonal therapy or SRS [12]. Here, we could demonstrate that reported changes of sexual orientation are not particularly associated with any transition event. Thus our data challenge the view that either hormonal therapy or SRS or any other event has a direct influence on self-reported sexual orientation.

### General characteristics

In the MtF group, most participants indicated that they were gynephilic (51%) or androphilic (26 %) before any change in sexual orientation might have occurred. In the FtM group most participants were gynephilic (73 %). These figures are in accordance with the historical and early reports from Magnus Hirschfeld [16] and recent reports [17]. Most of our participants reported on an early age of onset, regardless of being FtM or MtF. However, FtM started their transition process, including first psychological counseling, start of "real-life experience" in the new gender role, hormone treatment and sex reassignment surgery at a younger age than MtF. This is in accordance with the literature [18]. Others hypothesized that FtM in contrast to MtF feel lower social pressure in terms of parental disapproval of cross gender expression [19]–[21]. This may also explain for the discrepancy in latency between age of onset and first counseling for initiation of transition in MtF vs. FtM. Hypothesizing that gender dysphoric girls were more likely to be accepted by their family members, threshold for seeking medical advice could be lower. In contrast, cross-gender expression in boys is poorly accepted and thus referral to gender specialists happens earlier. This could also imply that FtM would also report changes in sex orientation less likely than MtF, since they did not have to suppress - consciously or unconsciously- their “initial” sexual orientation due to social pressure. Therefore a consecutively reported “change” would be less likely to occur.

### Change in self-reported sexual orientation

We could demonstrate that self-reported change in sexual orientation is quite common in MtF as well as FtM. 33 % of MtF and 22 % of FtM in our sample reported a change of their sexual orientation once in their life, a difference that was not significant. This is in accordance with earlier reports in MtF [11] and FtM [12]. Lawrence (2005) reported that up to 63 % of MtF experienced changes concerning their sexual orientation following sex reassignment surgery. However, Lawrence applied the more graduated Kinsey Scale Rating to determine sexual orientation, which is using intermediate scales such as “mostly attracted to”, “exclusively attracted to” et cetera. Interestingly, she found that there is little difference regarding preoperative characteristics between those who reported a change in sexual orientation following SRS and those who did not. Thus both groups could not be distinguished by any variables. This was similar in our study. We could not demonstrate a particular variable concerning transition process which would predict change in sexual orientation. Meier et al. [12] demonstrated that gynephilic FtM were more likely to report a change in sexual orientation after transition [12]. Contrastingly, in the present study sample the degree of FtM who reported a change in sexual orientation after transition was higher in androphilic FtM. It was hypothesized before that natal women have a more fluid sexual orientation compared to natal men in the general population. However, data on this topic are still sparse [23]–[24]. In natal men sexual orientation seems to be equivalent with sexual arousal that can be measured with penis plethysmography [23]. In line, MtF show specific sexual arousal patterns using vaginal photoplethysmography in surgically constructed neovaginas [25]. In contrast, natal women have genital arousal patterns that are not category specific (e.g. androphilia or gynephilia) but rather appear bisexual [23] and thus female sexual orientation was suggested to be more fluid and malleable. Yet plethysmography studies ignore cognitive components of sexual orientation that have an important role in self-reported sexual orientation (especially in women due to the ambiguous arousal patterns).

In heterosexual ( = gynephilic) men and heterosexual ( = androphilic) women only 4–5% report a change in sexual orientation during their lifetime. Changes in sexual orientation seem to be most common in bisexual subjects of both sexes of whom up to 77% report on such a change, primarily originating from a heterosexual orientation [22]. In homosexual ( = androphilic) men a change in sexual orientation occurs in about one third of adult subjects and is less common than in homosexual ( = gynephilic) women who report on change in sexual orientation in about two third of cases [26]. It is also important to stress that in homosexual persons bisexuality seems to be part of their coming out process [26].

We also regarded only those as a subgroup who had undergone the complete physical transition process. Here, most reported a change in sexual orientation following the surgical procedure in line with the study by Lawrence (2005). However, a relatively large proportion of participants (17.4% in MtF, 20% in FtM) reported on a change in sexual orientation without having undergone sex reassignment surgery or even before cross-sex hormone treatment challenging a relation between SRS and sexual orientation. Many transsexual persons may have suggested that a change in sexual orientation occurred after SRS because it demarcates an important life event and is often the last of many steps toward the development of the desired sex. Lawrence hypothesized in this context that SRS “*merely sets the stage for more confident misinterpretation and misreporting of an underlying sexual orientation that not only remains unchanged but is, in fact, unchangeable*” (p. 141 in [25]). We feel that the present existing data are not sufficient to decide whether or not sexual orientation can change in the course of life.

Meier and colleagues [12] reported a significant association of testosterone treatment in FtM and change in sexual orientation, however, in logistic regression analysis this was not independent of pre-transition sexual orientation. In our sample, initiation of cross-sex hormone therapy was not a significant predictor for change, but admittedly we had lower statistical power in our study. In addition it should be kept in mind that the Meier et al.-sample was based on online surveys which might have increased diversity of the participants in contrast to our clinical cohort. Five MtF and two FtM had not received cross-sex hormone treatment before change in sexual orientation. This highlights that self-reported change in sexual orientation can manifest independently of cross-sex hormone treatment.

### Changes of sexual orientation in MtF transsexual persons

MtF transsexual persons can be classified by age of onset or sexual orientation [27]. Following Ray Blanchard's sexual orientation typology [27], [28] two distinctly different types of MtF transsexual persons, namely homosexual (referred to as androphilic MtF in the present paper) and nonhomosexual MtF (gynephilic, bisexual and analloerotic MtF in the present paper) can be distinguished. Both groups differ profoundly: Androphilic MtF usually behave and identify as girls from earliest childhood which is reflected in female-typical toys, activities and playmates and later in female-typical occupations and hobbies [25]. Cross-dressing in this group of MtF is not associated with sexual arousal.

On the other hand, nonandrophilic MtF usually resemble ordinary cisgendered ( = the individual gender experience matches with the natal sex) men in their childhood activities and occupations [25], yet they intensely desire to be female. They also cross-dress, however, it evokes sexual arousal. Blanchard showed that androphilic MtF transsexual persons present with significantly higher cross-gender wishes and therefore seek treatment at significantly younger age [27]. In line, in the present study androphilic MtF sought psychological counseling earlier than nonandrophilic MtF.

Nonandrophilic MtF were also termed *autogynephilic* transsexual persons by Ray Blanchard [28], highlighting that an autogynephilic sexual orientation influences their cross-gender wishes (for a controversial statements see e.g. [29], [30] and an extensive description see [25]). Autogynephilic MtF transsexual persons are sexually oriented “toward the thought or image of themselves as women” [31]. Autogynephilia was thus proposed to represent an erotic target location error [25], [32]. Autogynephilic MtF transsexual persons often report the fantasy of sexual intercourse as a woman with a man, that was repeatedly described as faceless and abstract [25]. Yet this *pseudoandrophilia* has to be distinguished from genuine androphilia or homosexuality in MtF, or as Blanchard points it: “*the effective erotic stimulus, however, is not the male physique per se, as it is in true homosexual attraction, but rather the thought of being a female, which is symbolized in the fantasy of being penetrated by a male. For these persons, the imagined – occasionally real – male sexual partner serves the same function as women's apparel or makeup, namely, to aid and intensify the fantasy of being a woman*” [27]. Similarly, one of our participants that formally reported a change of sexual orientation from gynephilia towards androphilia stressed that “*I always wanted to experience sexual intercourse as a woman but I did not know what to do with my male body before the hormone treatment. I hated male bodies in general before*”. In this case a reported change in sexual orientation from gynephilic to androphilic can be attributed to autogynephilic fantasies. Another participant made clear that “*at the beginning I was not quite sure about my sexual orientation, but after one and a half years following sex reassignment surgery and after having had my first sexual intercourse with a man, I was able to love a man*.” Thus romantic love towards men followed sexual intercourse with men in this participant. In some way this narrative resembles that of some adolescent cissexual persons that encounter a similar confusion and uncertainty during puberty [33].

Moreover it is thinkable that some formerly nonandrophilic MtF transsexual persons only formally change their sexual orientation in the course of the transition because androphilia is socially more desirable for MtF transsexual persons. The socially desirability of androphilia in MtF was demonstrated in a recent study by Timo Nieder and colleagues (2011) where more than half of MtF transsexual persons reported that they were exclusively attracted to men, while the clinicians found it to be true only in about 10 % [34]. Kenneth Zucker and colleagues [34] demonstrated that even in adolescent boys with transvestism only 50 % admitted autogynephilic tendencies. In line, among gynephilic MtF transsexual persons we found the highest rate of reported change in sexual orientation (41.7 %). Considering the (even unintentional) socially desirable responding in terms of sexual orientation and autogynephilic fantasies, we hypothesize that this high figure overestimates the number of participants with a genuine change in sexual orientation. Participants may have reported on gynephilia at a time when they still had a male appearance and later changed their orientation towards the more accepted androphilia or bisexuality as MtF transsexual persons. Others would argue that the core sexual orientation in these participants was autogynephilia before and after transition [25]. This hypothesis is not totally supported by our data because none of the participants stated “other” sexual orientation like orientation towards other transsexual persons or autogynephilia although participants had the possibility to define their sexual orientation as “other” (with empty space to define). However, it is known that autogynephilia is often not reported or known, therefore in future studies we will explicitly ask for autogynephilic arousal.

The importance of individual experiences in the change of sexual orientation was also stressed by some participants. One androphilic MtF changed her sexual orientation towards bisexuality and explained: “*The change in my sexual attraction is part of my biography. I had experienced a lot of violence through men, so I was looking for a way out. My attraction may be more a matter of mind than of heart*.” This statement clarifies that there was no hormonal or biological mechanism that promoted her change of orientation but merely negative experiences. Another initially gynephilic MtF had a comparable explanation: “*Sexual desire decreased with hormone treatment. That I turned away from women as sexual partners has certainly also to do with my biography. I had experienced a lot of reactions that hurt me.*” It seems that the reduced libido through hormonal treatment in combination with negative experiences enabled the participant to engage in a relation with the other sex. Individual experiences also explained for the development of analloerotica following transitioning: “*I was repeatedly disappointed by interpersonal relations so I finally developed a disinterest in other people*”. Libido loss not only in relation to hormonal treatment but the surgical procedure was also reported: “*After the sex reassignment surgery I completely lost my libido*.” These qualitative data reveal that there seems to be not one common reason for a change in sexual orientation in MtF. A quantitative analysis of sexual orientation is thus flawed by unreported autogynephilia, individual biographical experiences, confusion about simultaneous experiences of andro- and gynephilic attraction and social factors that may prevent participants from reporting homosexuality or autogynephilia. *Reported* sexual orientation therefore seems to be influenced by a plethora of factors and may even be affected by personal decision, as one participant said: “*While some people think that gender identity is something you acquire or learn I think this was rather true for my alleged sexual orientation*”.

### Changes of sexual orientation in FtM transsexual persons

Gynephilic MtF and androphilic FtM were most likely to report a change in their sexual orientation. We argued before that autogynephilic fantasies may have influenced the high ratio of change in gynephilic MtF. So, what about androphilic FtM transsexual persons? Is correspondingly autoandrophilia a reason for the frequent change in androphilic FtM? Indeed we observed a surprisingly high ratio of change from androphilia to gynephilia in 66 % of androphilic FtM. But are androphilic FtM – which also represent “non classical” transsexual persons –sexually aroused by the thought of themselves as man, too? Until now there is limited data on autoandrophilia in FtM. However, autogynephilia was not reported for decades, too, until Ray Blanchard described the phenomenon [28]. We found several autoandrophilic narratives browsing web pages or simply googling the term. A recent qualitative study by Rowniak and Chesla [35] found indications for autoandrophilic erotic fantasies in androphilic FtM. They interviewed several FtM transsexual persons and one participant stressed that in the development of his own sexuality with his husband “*the interesting part is being sexual with him [the husband] as a man, was much better than being sexual with him as a woman, even though the act was pretty much the same*” (p. 453, [35]). Others described that sex with gay men was the strongest validation of being male [36]. Similarly, Schleifer concluded that sex with man reinforced masculinity of androphilic FtM and thus validates them as man [37]. However, in a qualitative analysis of interviews with androphilic FtM by Bockting, Benner and Coleman only 22 % of the participants reported sexual arousal in response to cross-dressing [38]. Since there exists almost no scientific literature concerning autoandrophilic erotic arousal in women or FtM transsexual persons, a systematic evaluation of this phenomenon would be of interest for upcoming studies. MtF autogynephilic transsexual persons manifest later and one reason is that some of them doubt whether they are “really transsexual” [25] because of sexual arousal related e.g. to cross-dressing. This has to be studied for autoandrophilic FtM transsexual persons as well.

Smith et al. [39] found no significant difference regarding GID symptoms in childhood between gynephilic and nongynephilic FtM, while other authors [40], who had divided age of onset in early childhood and post-pubertal onset, reported that early onset FtM were always sexually attracted to females. Levine and Lothstein [41] further found that in the majority of cases, early onset gender dysphoria was associated with a gynephilic orientation. Thus from the literature there seems a trend that androphilic MtF and gynephilic FtM start transitioning earlier.

Another pattern described by Rowniak and Chesla [35] was that some FtM participants “experienced gender dysphoria to such an extent at the time of adolescence and later that a comfortable and natural sexual activity was impossible” (p. 453). In line, one participant in the present study explained that “testosterone increased my libido. I would interpret my change in sexual orientation as having been confused before. Before mastectomy, I envied men and I rejected women, since I was in the wrong body. As soon as the distracting breast had been removed, I realized that I was into women.”

Another possible mechanism that acts on sexual orientation in FtM is testosterone treatment that stimulates libido in general [42]. In the present study we found no relation between onset of testosterone treatment and reported change of sexual orientation. In line, in former reports a possible relation was rather vague and profoundly differed in intervals between onset of testosterone treatment and reported change in sexual orientation ranging between 6 month and 7 years [35], [36]. Similarly some of our FtM participants described a subjective influence of testosterone treatment on sexuality but admitted other important factors in the change of sexual orientation: “*With hormone treatment, my libido increased and I felt more attracted to women, but this may also be connected with social pressure or what is regarded “right” defied by our society*”. We have discussed heterosexual behavior as socially desirable in MtF transsexual persons above and it thus seems also to play a role in FtM transsexual persons. Rowniak and Chesla [35] proposed that testosterone validates FtM transsexual persons' male gender identity and thus promotes the possibility to express their sexuality. Some participants supported this idea e.g. “*I think that testosterone influenced my biological body and promoted the change*”.

In gynephilic FtM a reported change of sexual orientation was less frequent. Six gynephilic FtM reported a change of sexual orientation towards bisexuality and androphilia in the present study. This may in part be explained by the fact that androphilic sexual behavior is complicated for FtM. Sex with male partners can induce intense gender dysphoria by being penetrated as a woman although feeling as a man. One participant in the study of Rowniak and Chesla stated that he didn't like being “feminized in bed” and others used the description that they were unable to have sex with men “until they were a man” [35]. Thus in these 6 participants androphilia may have been the original sexual orientation that became possible only after transitioning. In this case we wouldn't expect a genuine change of sexual orientation in these gynephilic FtM transsexual persons.

In conclusion we found a high degree of change in self-reported sexual orientation in gynephilic MtF and androphilic FtM. However, some of these participants may have been neither gynephilic nor androphilic but autogynephilic or autoandrophilic. In androphilic MtF and gynephilic FtM, a change in self-reported sexual orientation was less common and might also have been influenced by pre-transitioning dysphoria and uncertainty. The high ratios of change in self-reported sexual orientation – irrespective of whether these changes represent genuine changes – highlight the importance of this topic in transitioning especially for those transsexual persons that start transition with a partner. Changes in sexual orientation during and after transition bear the risk for partnered transsexual persons to lose a stable relationship with a spouse and may thus further increase the emotional burden of transsexual persons [43]. The questions: *Is a genuine change in sexual orientation really possible?* and *Is it more common in transsexual persons?* remain however still unanswered, leading us to other limitations of the present study:

### Limitations and future prospects

The present study is basically descriptive and explorative; hence there are some methodological limitations to be kept in mind. As every retrospective study, relying on self-reported variables our data are influenced by the recall-bias. In addition it has been shown that self-reported sexual orientation and sexual arousal patterns may diverge in transsexual persons [44]. To objectify sexual orientation aside from self-report, sexual arousal by means of penile or vaginal plethysmography [44] visual methods [45] or brain imaging [46] have been used in the past. A combination of qualitative narratives and biological methods (plethysmography and functional brain imaging) is needed to further elucidate the riddle whether sexual orientation can change in the general population and in transsexual persons. Such a study should be performed longitudinally to assess sexual arousal patterns before and after change of sexual orientation. In general, suggested biological underpinnings of transsexualism have so far been limited and debatable [47]–[49]. Therefore we need further research about biological and psychological reasons and consequences (like long-term effects of hormone therapy) of transsexualism in the future.

Self-reported sexual orientation studies have further been reported to be interfered by the fact that some persons do not answer the question truthfully [50]. Some transsexual people for example may want to present themselves as particular feminine (MtF) or masculine (FtM) and thus “classical” transsexual persons. Participants in the present study might have biased their reports on purpose or unwittingly towards a more gender-typical presentation [30]. This may also involve worries on denial of sex reassignment surgery. We feel that attempts to minimize such worries are important in future studies. We also suggest that researchers should explicitly ask for autogynephilic and autoandrophilic sexual orientation.

Finally the results may have been biased by the fact that there are some transsexual persons who do not want to complete physical transition and we did not discriminate between those not having received cross-sex hormone treatment or surgery and those not wanting to.

A strength of our study is that the investigated group was homogenous in regard to ethnicity and had experienced similar treatment modalities due to the single center design. Furthermore in Germany sex reassignment surgery is generally reimbursed by public or private health insurance if the diagnosis GID is settled and no individual in our study would have to forego sex reassignment due to financial reasons. In addition the response rate was quite high (> 95 %), minimizing selection bias and increasing its representation. The combination of quantitative data with the free text module concerning change in SO enriches current research, since most of the other studies that investigated SO in transsexual persons either had large samples and no free text information [12] or only small samples but clinical interviews [9], [35] or were only studying a subgroup of transsexual persons [11].

## Conclusion

By collecting quantitative and qualitative data in a large sample of transsexual persons, we demonstrate that self-reported change in sexual orientation is a common phenomenon in transsexual persons. Transition was not directly involved in this change, since a significant number of participants reported a change in sexual orientation prior to first psychological counseling and prior to initiation of cross-sex hormone treatment. The participants provided diverse individual explanation models, revealing that personal history, social environment as well as autoerotic feelings may impact on a change in sexual orientation.

## Supporting Information

Text S1 **All Quotes from the participants regarding self-reported change in sexual orientation.**
(DOCX)
