# Supplementary material for: Transgender Transitioning and Change of Self-Reported Sexual Orientation
Source: PLoS One. 2014 Oct 9;9(10):e110016. doi: 10.1371/journal.pone.0110016 (PMC4192544; doi:10.1371/journal.pone.0110016)
Supplement: Text S1 — All Quotes from the participants regarding self-reported change in sexual orientation. (DOCX) [file pone.0110016.s001.docx]

Text S1: Quotes from the participants regarding self-reported change in sexual orientation

**MTF**

G-B “At the beginning I was not quite sure about my sexual orientation, but after one and a half years following sex reassignment surgery and after having had my first sexual intercourse with a man, I was able to love a man.”

“Simultaneously to initiation of cross-sex hormone therapy, I felt a change in my sexual attraction, but it may also have something to do with the fact that I accepted being transgender then”

“I experienced a change in sexual attraction three years following the sex

reassignment surgery”

G-A “At the time when I was able to accept my inner feelings my sexual orientation changed. I think that initiation of cross-sex hormone treatment was decisive in this regard, too “

“Sexual desire decreased with hormone treatment. That I turned away from women as sexual partners has certainly also to do with my biography. I had experienced a lot of reactions that hurt me.”

While some people think that gender identity is something you acquire or learn I think this was rather true for my alleged sexual orientation”

„The hormone treatment significantly shifted my sexual orientation towards men”

“I don’t think that the hormone treatment itself changed my sexual orientation, but by completing my transition, I felt more attracted to men than to women”

“I always wanted to experience sexual intercourse as a women but I did not know what to do with my male body before the hormone treatment. I hated male bodies in general before.”

AN-B “Before the cross-sex hormone treatment I was neither interested in

women nor in men”

“I did not know anything about transgender people at this time and I felt too depressed to think about sex or partnership at all but now I live in a relationship with a man.”

A-B “The change in my sexual attraction is part of my biography. I had experienced a lot of violence through men, so I was looking for a way out. So my attraction may be more a matter of mind than of heart.”

G-AN “I was repeatedly disappointed by interpersonal relations so I finally

experienced a disinterest in other people”

“After the sex reassignment surgery I completely lost my libido.”

A-G “I do not feel that there is any connection between the surgery or the hormone treatments in regard to my change in sexual attraction”

**FTM**

A-G “Testosterone increased my libido. I would interpret my change in sexual orientation as having been confused before. Before mastectomy, I envied men and I rejected women, since I was in the wrong body. As soon as the distracting breast had been removed, I realized that I was into women.”

“With hormone treatment, my libido increased und felt more attracted to women, but this may also be connected with social pressure or what is regarded “right” defied by our society.”

“During adolescence I wanted to have contacts to men, since it was

regarded normal, when I turned 25 I accepted being “lesbian” and with

start of hormone treatment I felt attracted to women.”

G-A “Since initiation of hormone treatment, I feel more attracted to men.”

G-B I think that testosterone influenced my biological body and promoted the change.

*G-B = gynephilic to bisexual, G-A = gynephilic to androphilic, AN-B = analloerotic to bisexual, A-B = androphilic to bisexual, G-AN = gynephilic to analloerotic, A-G= androphilic to gynephilic, G-B = gynephilic to bisexual*
